# The effect of using a large language model to respond to patient messages

**DOI:** 10.1016/S2589-7500(24)00060-8

**Published:** 2024-04-24

**Authors:** Shan Chen, Marco Guevara, Shalini Moningi, Frank Hoebers, Hesham Elhalawani, Benjamin H Kann, Fallon E Chipidza, Jonathan Leeman, Hugo J W L Aerts, Timothy Miller, Guergana K Savova, Jack Gallifant, Leo A Celi, Raymond H Mak, Maryam Lustberg, Majid Afshar, Danielle S Bitterman

**Affiliations:** Artificial Intelligence in Medicine Program, Mass General Brigham, Harvard Medical School, Boston, MA, USA; Department of Radiation Oncology, Brigham and Women’s Hospital and Dana-Farber Cancer Institute, Boston, MA 02115, USA; Computational Health Informatics Program, Boston Children’s Hospital, Harvard Medical School, Boston, MA, USA; Artificial Intelligence in Medicine Program, Mass General Brigham, Harvard Medical School, Boston, MA, USA; Department of Radiation Oncology, Brigham and Women’s Hospital and Dana-Farber Cancer Institute, Boston, MA 02115, USA; Department of Radiation Oncology, Brigham and Women’s Hospital and Dana-Farber Cancer Institute, Boston, MA 02115, USA; Artificial Intelligence in Medicine Program, Mass General Brigham, Harvard Medical School, Boston, MA, USA; Department of Radiation Oncology, Brigham and Women’s Hospital and Dana-Farber Cancer Institute, Boston, MA 02115, USA; Department of Radiation Oncology, GROW School for Oncology and Reproduction, Maastricht University, Maastricht, Netherlands; Department of Radiation Oncology, Brigham and Women’s Hospital and Dana-Farber Cancer Institute, Boston, MA 02115, USA; Artificial Intelligence in Medicine Program, Mass General Brigham, Harvard Medical School, Boston, MA, USA; Department of Radiation Oncology, Brigham and Women’s Hospital and Dana-Farber Cancer Institute, Boston, MA 02115, USA; Department of Radiation Oncology, Brigham and Women’s Hospital and Dana-Farber Cancer Institute, Boston, MA 02115, USA; Department of Radiation Oncology, Brigham and Women’s Hospital and Dana-Farber Cancer Institute, Boston, MA 02115, USA; Artificial Intelligence in Medicine Program, Mass General Brigham, Harvard Medical School, Boston, MA, USA; Department of Radiation Oncology, Brigham and Women’s Hospital and Dana-Farber Cancer Institute, Boston, MA 02115, USA; Radiology and Nuclear Medicine, GROW and Cardiovascular Research Institute Maastricht, Maastricht University, Maastricht, Netherlands; Computational Health Informatics Program, Boston Children’s Hospital, Harvard Medical School, Boston, MA, USA; Computational Health Informatics Program, Boston Children’s Hospital, Harvard Medical School, Boston, MA, USA; Laboratory for Computational Physiology, Massachusetts Institute of Technology, Cambridge, MA, USA; Laboratory for Computational Physiology, Massachusetts Institute of Technology, Cambridge, MA, USA; Division of Pulmonary, Critical Care and Sleep Medicine, Beth Israel Deaconess Medical Center, Boston, MA, USA; Department of Biostatistics, Harvard T H Chan School of Public Health, Boston, MA, USA; Artificial Intelligence in Medicine Program, Mass General Brigham, Harvard Medical School, Boston, MA, USA; Department of Radiation Oncology, Brigham and Women’s Hospital and Dana-Farber Cancer Institute, Boston, MA 02115, USA; Department of Medical Oncology, Yale School of Medicine, New Haven, CT, USA; Department of Medicine, University of Wisconsin School of Medicine and Public Health, Madison, WI, USA; Artificial Intelligence in Medicine Program, Mass General Brigham, Harvard Medical School, Boston, MA, USA; Department of Radiation Oncology, Brigham and Women’s Hospital and Dana-Farber Cancer Institute, Boston, MA 02115, USA; Computational Health Informatics Program, Boston Children’s Hospital, Harvard Medical School, Boston, MA, USA

The relentless increase in administrative responsibilities, amplified by electronic health record (EHR) systems, has diverted clinician attention from direct patient care, fuelling burnout.^1^ In response, large language models (LLMs) are being adopted to streamline clinical and administrative tasks. Notably, Epic is currently leveraging OpenAI’s ChatGPT models, including GPT-4, for electronic messaging via online portals.^2^ The volume of patient portal messaging has escalated in the past 5–10 years,^3^ and general-purpose LLMs are being deployed to manage this burden. Their use in drafting responses to patient messages is one of the earliest applications of LLMs in EHRs.^2^

Previous works have evaluated the quality of LLMs responses to biomedical and clinical knowledge questions.^4-6^ However, the ability of LLMs to improve efficiency and reduce cognitive burden has not been established, and the effect of LLMs on clinical decision making is unknown. To begin to bridge this knowledge gap, we carried out a proof-of-concept end-user study assessing the effect and safety of LLM-assisted patient messaging. This study serves as a call to action for a measured approach to implementing LLMs within EHRs, including evaluations that reflect how they will actually be used in clinical settings and considerations of human factors.^7^

In this two-stage observational study was conducted in 2023 at Brigham and Women’s Hospital, Boston, MA, USA, we sought to understand how LLM assistance for electronic patient portal messaging in EHRs (ie, using an LLM to draft a response for a clinician to edit) might impact subjective efficiency, clinical recommendations, and potential harms. The overall study schema is in the appendix (p 1).

GPT-4 was prompted with few-shot exemplars to generate 100 scenario and symptom question pairs for patients with cancer. This content was manually reviewed and edited by an oncologist (DSB) to ensure that they reflected a realistic clinical picture. Separately, GPT-4 was prompted to generate a response to the patient’s question. The prompting approaches are in the appendix (p 2).

Six board-certified attending radiation oncologists (SM, FH, HE, BHK, FEC, and JL) were first asked to respond to the patient messages as they normally would in clinical practice (manual responses; stage 1), and then asked to edit the GPT-4 responses (LLM drafts) so that they were clinically acceptable responses to send a patient (LLM-assisted responses; stage 2). The effect of LLM assistance on patient messaging was evaluated by surveys evaluating quality, safety, and helpfulness, and content analysis of responses. Each physician evaluated 26 scenario and message pairs in both stages, yielding 56 dual-annotated cases and 44 single-annotated cases. The physicians were masked to the source of the messages. Examples of how the scenarios and surveys were presented along with instructions and real responses are in the appendix (pp 3-7).

To evaluate differences in the content of responses generated in stage 1 and stage 2 (manual, LLM draft, and LLM-assisted responses), guidelines were created to annotate ten content categories (appendix p 8). 50 responses were dual-annotated by content-based categorical evaluation by two physicians who did not participate in stage 1 or stage 2 of the study (DSB and MA); Cohen’s kappa was 0·75 or more for all categories. The remaining responses were single annotated by DSB.

Statistical analyses were carried out using the statistical Python package in SciPy v1.10.1. All pairwise comparisons were done using the Mann–Whitney *U* test. p of less than 0·05 were considered statistically significant. All OpenAI application programming interface settings for responses were set to temperature=0 and Top_p=0. This study was approved by the Dana-Farber/Harvard Cancer Center Institutional Review Board.

The mean manual response (34 words) was shorter than the LLM draft (169 words) and LLM-assisted responses (160 words; p<0·0001 for all comparisons). The full stage 1 and 2 survey results are in the appendix (p 12). It was felt by the assessing physicians that the LLM drafts posed a risk of severe harm in 11 (7·1%) of 156 survey responses, and death in one (0·6%) survey response. The majority of harmful responses were due to incorrectly determining or conveying the acuity of the scenario and recommended action (appendix p 19). The assessing physicians reported that the LLM draft improved subjective efficiency in 120 (76·9%) of 156 cases.

Interphysician agreement in the clinical content of responses was poor for manual responses (mean Cohen’s kappa 0·10) but improved with LLM-assistance (mean Cohen’s kappa 0·52).

The content of the LLM-assisted responses were more similar to the LLM drafts (p=0·81) than to manual responses (p<0·0001; figure). Compared with manual responses, LLM drafts were less likely to include content on direct clinical action, including instructing patients to present urgently or non-urgently for evaluation, and to describe an action the clinician will take in response to the question (p<0·0001 for all); but more likely to provide extensive education, self-management recommendations, and a contingency plan (p<0·0001 for all).

Our findings show how LLM assistance might offer a so-called best of both worlds scenario, simultaneously reducing physician workload, improving consistency across physician responses, and enhancing the informativeness and educational value of responses. The quality of the additional LLM-generated content is noteworthy, because LLM drafts were generally acceptable and posed minimal risk of harm.

Yet, we also showed that existing evaluations are insufficient to understand clinical utility and risks because LLMs might unexpectedly alter clinical decision making, and that physicians might use LLMs’ assessments instead of using LLM responses to facilitate the communication of their own assessments. LLMs might affect clinical decision making in ways that need to be monitored and mitigated when used in a human and machine collaborative framework. The content of physician responses changed when using LLM assistance, suggesting an automation bias and anchoring, which could have a downstream effect on patient outcomes. The improved interphysician agreement and similarity of response content between LLM drafts and LLM-assisted responses suggest that physicians might not simply use LLMs to better phrase their own assessment, but instead adopt the assessment by the LLM. This finding raises the question of the extent to which LLM assistance is decision support versus LLM-based decision making. Additionally, a minority of LLM drafts, if left unedited, could lead to severe harm or death. Thus, there is a need for new approaches for evaluation and monitoring, especially as trust in LLMs builds and clinicians become less vigilant and more reliant on LLMs.^8^ In our study, harmful content was often associated with inadequate recognition or communication of the scenario’s acuity, rather than errors in biomedical knowledge. Assessments of encoded general biomedical knowledge, such as performance on medical board exams, are a first step toward clinical applications,^5^ but should not be used as surrogates for the clinical expertise and acumen needed to care for patients.

We showed that existing evaluations are insufficient to understand clinical utility and risks because LLMs might unexpectedly alter clinical decision making, and that physicians might use LLMs’ assessments instead of using LLM responses to facilitate the communication of their own assessments. Despite being a simulation study, these early findings provide a safety signal indicated a need to thoroughly evaluate LLMs in their intended clinical contexts, reflecting the precise task and level of human oversight.^9^ Moving forward, more transparency from EHR vendors and institutions about prompting methods are urgently needed for evaluations. LLM assistance is a promising avenue to reduce clinician workload but has implications that could have downstream effect on patient outcomes. This situation necessitates treating LLMs with the same rigor in evaluation as any other software as a medical device.^10^ Physicians and institutions must exercise caution as the health-care industry embraces these advanced technologies, because it is imperative to balance their innovative potential with a commitment to patient safety and care quality.

## Supplementary Material

Supplement

## Figures and Tables

**Figure:**
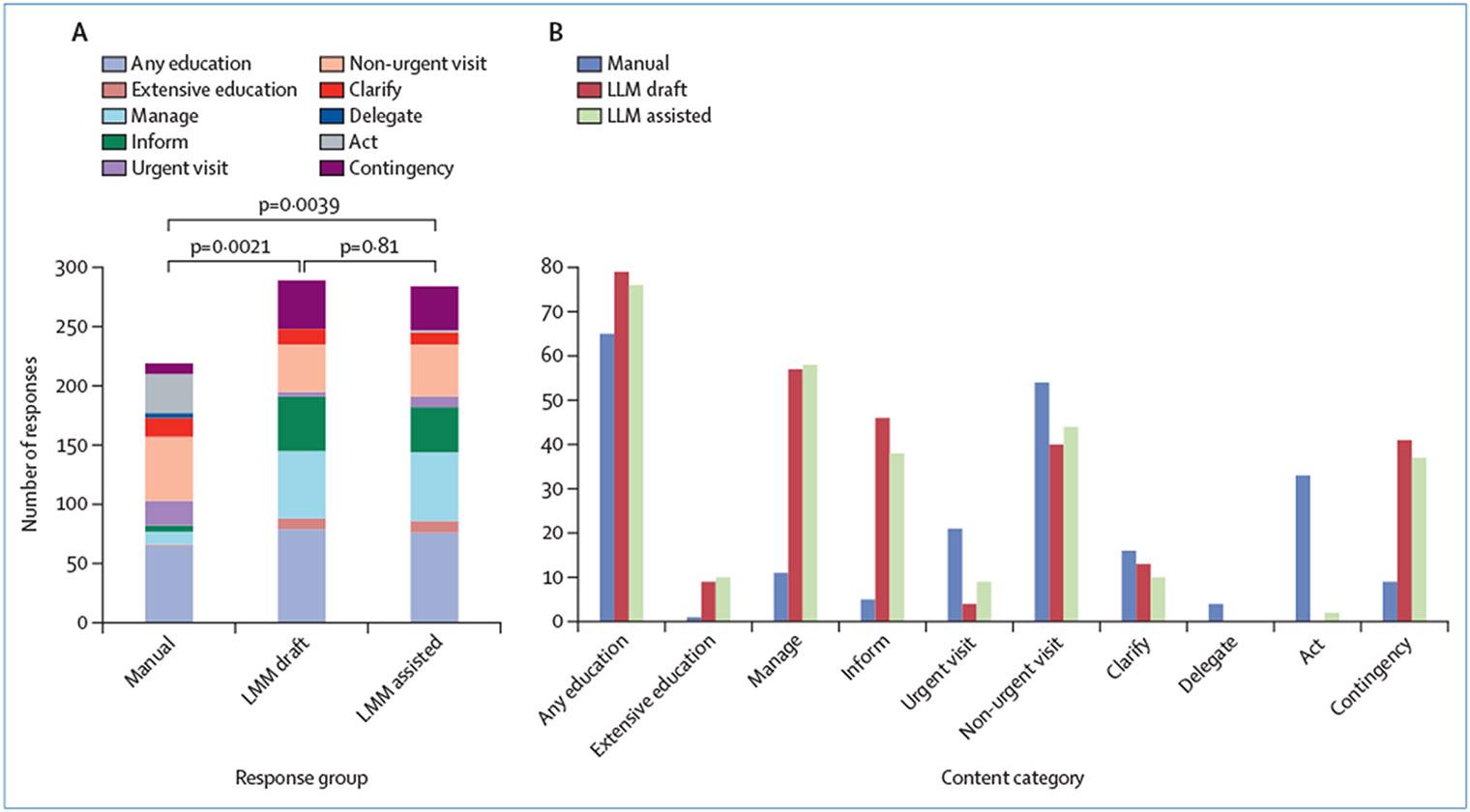
Response content comparisons Total number of responses that included each content category for manual, LLM draft, and LLM-assisted responses. (A) The overall distribution of content categories present in each response type. Pairwise comparisons of the overall distributions according to response type were done using Mann–Whitney U tests. (B) Visualisation of the total count of each category for the three response types. LLM=large language model.
